# Drought response revealed by chromatin organization variation and transcriptional regulation in cotton

**DOI:** 10.1186/s12915-024-01906-0

**Published:** 2024-05-20

**Authors:** Boyang Zhang, Yuexuan Long, Liuling Pei, Xianhui Huang, Baoqi Li, Bei Han, Mengmeng Zhang, Keith Lindsey, Xianlong Zhang, Maojun Wang, Xiyan Yang

**Affiliations:** 1 National Key Laboratory of Crop Genetic Improvement, Hubei Hongshan Laboratory, Huazhong Agricultural University, Wuhan, China; 2 Department of Biosciences, Durham University, South Road, Durham, DH1 3LE UK

**Keywords:** Cotton, Drought stress response, Gene expression, Subgenomic expression bias, TAD

## Abstract

**Background:**

Cotton is a major world cash crop and an important source of natural fiber, oil, and protein. Drought stress is becoming a restrictive factor affecting cotton production. To facilitate the development of drought-tolerant cotton varieties, it is necessary to study the molecular mechanism of drought stress response by exploring key drought-resistant genes and related regulatory factors.

**Results:**

In this study, two cotton varieties, ZY007 (drought-sensitive) and ZY168 (drought-tolerant), showing obvious phenotypic differences under drought stress, were selected. A total of 25,898 drought-induced genes were identified, exhibiting significant enrichment in pathways related to plant stress responses. Under drought induction, A_t_ subgenome expression bias was observed at the whole-genome level, which may be due to stronger inhibition of D_t_ subgenome expression. A gene co-expression module that was significantly associated with drought resistance was identified. About 90% of topologically associating domain (TAD) boundaries were stable, and 6613 TAD variation events were identified between the two varieties under drought. We identified 92 genes in ZY007 and 98 in ZY168 related to chromatin 3D structural variation and induced by drought stress. These genes are closely linked to the cotton response to drought stress through canonical hormone-responsive pathways, modulation of kinase and phosphatase activities, facilitation of calcium ion transport, and other related molecular mechanisms.

**Conclusions:**

These results lay a foundation for elucidating the molecular mechanism of the cotton drought response and provide important regulatory locus and gene resources for the future molecular breeding of drought-resistant cotton varieties.

**Supplementary Information:**

The online version contains supplementary material available at 10.1186/s12915-024-01906-0.

## Background

Approximately one-third of Earth’s land is arid or semi-arid, posing a significant challenge to global agriculture [1]. Global warming exacerbates this by reducing water resources, compounding drought effects. Drought stress has become the major limiting factor for global agricultural production, severely restricting the normal growth and development of plants [2, 3]. To cope with drought stress, plants have evolved multiple regulatory strategies, including reducing water loss, balancing water supply to important organs, and maintaining cell water content [4]. The effects of drought stress on plants are diverse, encompassing morphological characteristics, physiological metabolism, hormone secretion, and signal transduction [5]. In signal transduction pathways, transcription factors (TFs) bind to *cis*-regulatory elements (CREs) in the promoter region of stress-responsive genes, regulate the expression of downstream genes, and influence the plants adaptation to stress. During drought stress, TFs act alone or in coordination, forming complex regulatory networks [6]. Various TF families, including NAC, WRKY, ERF, MYB, and bZIP, have been shown to enhance plant drought tolerance in *Arabidopsis* [7]. Overall, a better understanding of the molecular mechanisms underlying plant drought resistance may help facilitate the development of crops that are more resilient to these environmental stresses, ultimately enhancing global food security.

Cotton is widely cultivated and provides a fiber source for human use [8]. Cotton is also an important food source, and cottonseed oil and protein have high economic value [9]. For a long time, drought stress has become the main limiting factor for cotton production. During drought stress, cotton activates its own protection system to maintain physiological water balance. For example, it increases the absorption of soil moisture by roots, reduces water loss by closing stomata, and regulates antioxidant and osmotic processes within tissues. Cotton can alleviate osmotic stress under drought by mechanisms such as accumulating and transporting inorganic ions and small molecule organic substances. As one of the most important stress response hormones, the analysis of abscisic acid (ABA) is involved in many studies aimed at improving cotton water use efficiency and drought resistance [10]. Additionally, several TFs related to drought stress signal transduction have been identified in cotton, such as GhWRKY41, GhWRKY59, and GhMYB108-like [11–13].

Eukaryotic genomes are organized into chromatin, a nuclear complex of DNA, RNA, and proteins [14, 15]. Three-dimensional (3D) genomics mainly studies the 3D spatial structure of the genome and its impact on gene expression and regulation. It utilizes high-throughput sequencing and chromosome conformation capture technologies to unveil chromatin’s organization, gene interactions, and their roles in DNA replication and transcription regulation [16]. Recent studies reveal a hierarchical structure in plant genomes, including chromatin territories, A/B compartments, topologically associating domains (TADs), and chromatin loops [17]. Dynamic changes of 3D chromatin structure are linked to gene regulation and plant physiology, especially under stress. In cold stress, rice genomes exhibit reduced long-range interactions, indicating disaggregation [18]. In terms of heat stress, it has been found that heat shock causes global rearrangement of the 3D genome in *Arabidopsis* [19]. Rice genomes show altered chromatin structures with A/B compartment switching, TAD size changes, and loss of proximal *cis*-interactions in response to heat stress [20]. Furthermore, these changes in chromatin structure are related to changes in chromatin accessibility and gene expression.

However, there have been no reports on how the 3D genome structure of cotton changes under drought stress and how important functional genes may be regulated by dynamic changes in chromatin structure. Here, we investigate the transcriptional regulation mechanism of cotton drought resistance by studying the transcriptome and chromatin organization of sensitive and tolerant cotton varieties under different drought conditions, drawing dynamic maps of the 3D genome, screening important drought-responsive genes regulated by dynamic variations in chromatin structure, and conducting functional analysis.

## Results

### Phenotypic differences under drought resistance of two cotton varieties

**Fig. 1:**
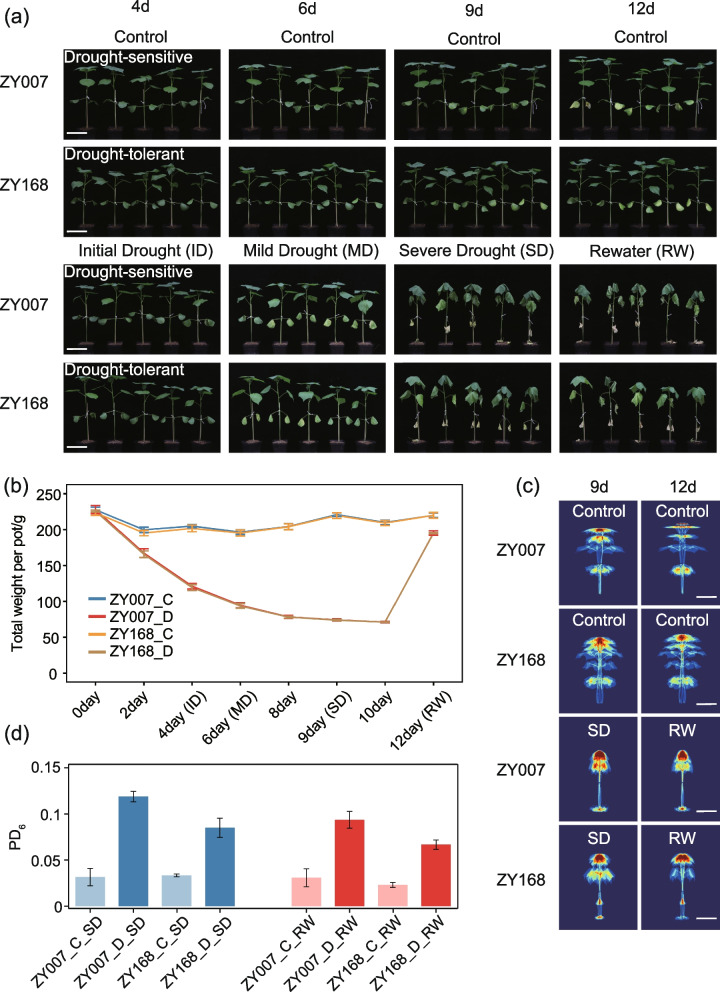
Phenotypes and i-trait (PD_6_) of drought-tolerant and drought-sensitive varieties’ seedlings under different drought stresses conditions. **a** The dynamic phenotypes of drought-tolerant and drought-sensitive variety seedlings under different drought treatments. ID (Initial Drought), MD (Mild Drought), and SD (Severe Drought) represent the fourth day, sixth day, and ninth day after drought stress, respectively. RW (re-water) represents the second day after re-watering. Scale bar is 10 cm. **b** Average weight per pot at different time-points after drought treatment. The gray lines represent the four time-points. C represents normal water supply condition; D represent drought stress condition, respectively. **c** Modeling color maps of PDs of drought-tolerant and drought-sensitive varieties under MD and SD. Scale bar is 10 cm. **d** PD_6_ of drought-tolerant and drought-sensitive varieties. Higher PD_6_ represents more severe wilting status

We selected two varieties, ZY007 (drought-sensitive) and ZY168 (drought-tolerant), that showed significant differences in performance under drought treatment, and observed their plant phenotypes at different stages of drought. On the fourth day of drought treatment (Initial Drought, ID), the water content of the treated group gradually decreased, and the edges of the leaves of both varieties softened slightly. On the sixth day of drought treatment (Mild Drought, MD), the water content of the treated group continued to decrease, and the leaves of both varieties wilted completely. On the ninth day of drought treatment (Severe Drought, SD), the water content of the treated group further decreased, and the true leaves of both varieties gradually wilted. At this time, the drought-tolerant variety ZY168 showed better growth than the drought-sensitive ZY007, as evidenced by more severe wilting of the leaves of ZY007. On the 11th day of drought treatment, the treated group was saturated with water, and after 24 h of watering (Re-water, RW), ZY168 gradually returned to normal, while ZY007 remained in a state of continued wilting (Fig. 1a,b).

At the same time, we created pseudocolor maps of the plants from both varieties under different drought conditions (Fig. 1c), and PD_6_ (Plant Density dimension 6) was determined to be a new indicator for plant drought severity. The higher the PD_6_, the more severe the wilting of the plant [21]. We found that under SD conditions, the PD_6_ of ZY007 was higher, indicating more severe wilting and higher sensitivity to drought. After rehydration, the PD_6_ of ZY168 decreased more, indicating a faster recovery from wilting and stronger post-drought resilience (Fig. 1d).

### Construction of drought-induced expression landscape in cotton

In order to explore the similarities and differences of the drought resistance of the two cotton varieties at the transcriptional level, we sampled the leaves of the drought-treated plants and control plants at the above four treatment stages (ID, MD, SD, and RW). A total of 32 samples were obtained (two biological replicates), and transcriptome sequencing was performed on the samples. After preliminary analysis, we collected a total of 2.88 billion valid reads (Additional file 2: Table S1), and the correlations between biological replicates based on reads count of the bins were between 0.87 and 0.96 (Additional file 1: Fig. S1a).

Subsequently, we calculated the expression levels of all genes and constructed an expression matrix. The correlations of gene expression between two biological replicates were between 0.96 and 0.99, which allowed us to merge two biological replicates (Additional file 1: Fig. S1b). Cluster analysis showed that all samples were grouped into four clusters. The first cluster included controls and the ID stage, the second cluster included controls and RW stage of ZY168, the third cluster included the MD stage, and the fourth cluster included the SD stage and the RW stage of ZY007 (Additional file 1: Fig. S1c). From these results, it can be inferred that different sampling time points under drought can be effectively distinguished at the transcript level. Moreover, at the transcript level, the RW samples of the drought-sensitive variety ZY007 still exhibited transcriptional expression similar to that of the SD stage samples, while the drought-tolerant variety ZY168 showed significant recovery in gene expression after rewatering, which was close to that of the ID stage.

Compared to the control, the number of expressed genes decreased with prolonged and intensified drought (Additional file 1: Fig. S2a). Subsequently, we conducted pairwise comparisons of control and drought-treated samples to identify differentially expressed genes (DEGs), resulting in 2619, 10,353, 19,460, and 17,582 DEGs in the four time points, respectively (Additional file 1: Fig. S2b; Additional file 2: Table S2). Except for the ID stage, the number of up-regulated genes was lower than that of down-regulated genes, indicating an overall decrease in gene expression levels under drought. We performed GO enrichment analysis on these DEGs, and the results showed significant enrichment in pathways related to plant stress response, such as ethylene, abscisic acid, jasmonic acid, kinase activity, water transport, and redox reactions (Additional file 1: Fig. S2c). These results illustrate a cotton expression profile in response to drought stress.

### Asymmetric expression of subgenomes under drought induction

**Fig. 2:**
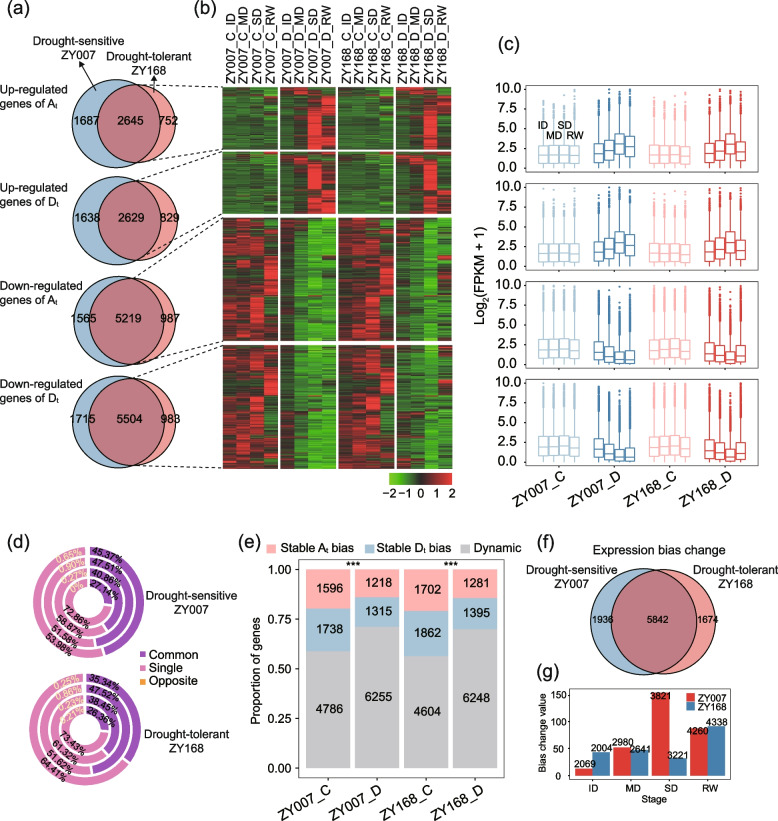
Expression patterns of drought-induced genes and expression preferences among homoeologous genes. **a** The number of drought-induced genes in the two varieties, which are divided into up/down-regulated and A_t_/D_t_ subgenomes. **b–c** Expression patterns of genes in the intersection part of the two varieties at different stages. **d** The genes were divided into three groups according to their expression direction of homoeologous genes in the two subgenomes under drought stress treatment compared to the control. **e** The numbers and proportions for the three types of homoeologous genes with bias expression under four conditions. Red, blue, and gray colors represent stable A_t_ bias expression, stable D_t_ bias expression, and dynamic bias expression, respectively (two-sided Chi-square test, ***P < 0.001). **f** Number of genes with changed expression bias induced by drought. **g** Genome-wide bias values for two varieties at four drought stress stages. C and D represent normal water supply and drought stress condition, respectively

To investigate the differences between two varieties in response to drought stress, we defined drought-induced genes (see “Methods”). The results showed that in ZY007, the gene expressions levels of a total of 22,606 genes were changed in response to drought, including 8601 up-regulated and 14,005 down-regulated genes. In ZY168, the gene expressions levels of a total of 19,552 genes were changed in response to drought, including 6857 up-regulated and 12,695 down-regulated genes (Fig. 2a). Analysis of the expression patterns of differentially expressed genes shared between the two varieties revealed that compared to ZY007, ZY168 exhibited stronger expression recovery ability after rehydration. Both up-regulated and down-regulated genes showed more expression-recovered trends after rehydration in ZY168 (Fig. 2b,c). After rehydration, the numbers of recovered and differentially expressed drought-induced genes were 8109 and 14,497 in ZY007 and 3326 and 16,226 in ZY168, respectively (Chi-squared test, *P* ≤ 2.2 × 10^–26^). This suggests that ZY168 has stronger recovery ability at the expression level than ZY007 consistent with phenotype.

As an allopolyploid crop, cotton exhibits a certain degree of asymmetry between subgenomes [22]. To investigate whether the response of the two subgenomes has asymmetry under drought, we performed the following three types of analysis. Firstly, we compared the expression changes of homoeologous genes in the two subgenomes relative to controls under drought treatment. Based on the direction of expression changes, the homoeologous genes were divided into three categories: common, single, and opposite (see “Methods”). The results show that the largest category was single, followed by common, and only a very small proportion (less than 0.9%) of homoeologous genes showed differential expression in opposite directions (Fig. 2d). Moreover, as the degree of drought intensifies, the proportion of single genes decreases while that of common genes increases. This trend is restored after rehydration. These findings suggest that under drought conditions, homoeologous genes exhibit convergent expression patterns.

Secondly, we compared the bias expression patterns of homoeologous genes during different stages. We identified a total of 23,703 subgenome homoeologous gene pairs. Among them, 8121 homoeologous gene pairs with bias expression of subgenome were identified in ZY007 under control conditions, 8789 in ZY007 under drought, 8169 in ZY168 under control conditions, and 8925 in ZY168 under drought. Analysis of the dynamic bias expression patterns of these homoeologous gene pairs during four stages (see “Methods”) showed that homoeologous gene pairs with dynamic bias expression between four stages accounted for the majority (Fig. 2e). Furthermore, the proportion of homoeologous gene pairs exhibiting dynamic bias expression increased significantly under drought and obviously decreased after rehydration in both varieties (Additional file 1: Fig. S2d).

Thirdly, to investigate the effects of drought stress on bias expression of subgenome, we identified 7778 and 7516 genes with expression preference changes induced by drought in ZY007 and ZY168, respectively (Fig. 2f). Approximately 62% of these genes were shared by both varieties. To further explore the differences between the two varieties, we defined a bias value (see “Methods”). Results from the calculation of the bias values for the whole genome at four stages indicated that the genome exhibited an expression bias of A_t_ subgenome under drought (Fig. 2g). The bias value gradually increased for ZY007 and showed a trend of first increasing and then decreasing for ZY168 (Fig. 2g). To investigate the cause of the bias expression of the A_t_ subgenome, we conducted detailed analysis of the gene expression levels. The results indicated that under drought stress, the expression levels of both subgenomes decreased, but the decrease was greater for the D_t_ subgenome (Additional file 1: Fig. S2e). This phenomenon was particularly evident of SD in ZY007, which corresponded to a significant increase in the bias value. These results indicate that with the increasing severity of drought, expression of the D_t_ subgenome was more strongly inhibited than A_t_ subgenome in drought-sensitive ZY007, while the inhibition difference of both subgenomes in ZY007 was greater than in ZY168.

### Construction of drought-related co-expression networks

**Fig. 3:**
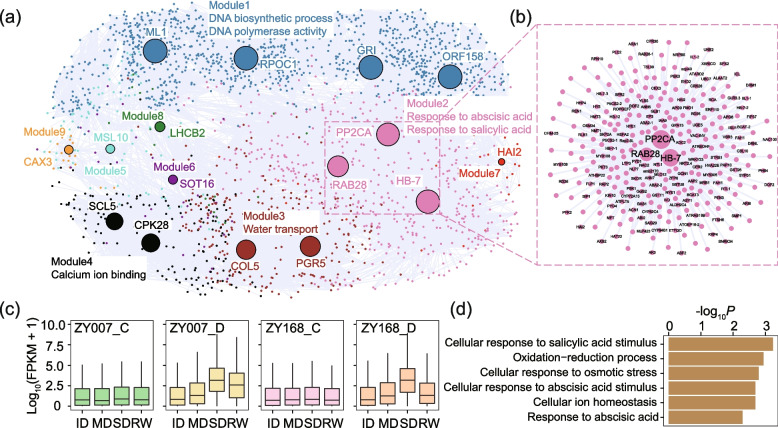
Co-expression network construction and screening. **a** The display of the co-expression network, different colors represent different modules, and the size of the hub gene is positively correlated with the sum of the weight values. **b** A detailed display of the connection of the three hub genes in module 2. **c** The expression patterns of the genes of module 2 in two varieties, two treatments, and four stages are shown. **d** GO enrichment results of genes in module 2

To further screen and identify drought-related genes from the transcriptome, we constructed a co-expression network of cotton in response to drought using the union of all DEGs, comprising 26,412 genes. A threshold weight value greater than 0.2 was used to filter the co-expression relationships, resulting in 2402 genes and 34,064 co-expression relationships in the network (Fig. 3a). The co-expression network consisted of nine modules, with each module containing a different number of genes (ranging from 7 to 1291). We then identified hub genes for each module by selecting genes with the highest total weight values (Fig. 3b). We further examined the expression patterns of genes in each module in response to drought in two varieties. As a result, we found that genes in module 2 showed significant upregulation under drought in both varieties, followed by obvious recovery after rehydration, with ZY168 exhibiting stronger recovery (Fig. 3c). We applied Gene Ontology (GO) enrichment analysis for each module and found that module 2 was enriched in pathways related to ion balance, jasmonic acid response, abscisic acid response, osmotic pressure regulation, and redox reactions, which are closely related to plant stress responses (Fig. 3d; Additional file 2: Table S3). Module 2 also contained many previously reported key genes related to drought resistance, such as *PP2CA* [23], *RAB28* [24], and *HB-7* [25] (Fig. 2b; Additional file 2: Table S4). Overall, module 2 is the co-expression module closely associated with drought stress, and its genes have close connections with drought resistance.

### A/B compartment switching under drought treatment

**Fig. 4:**
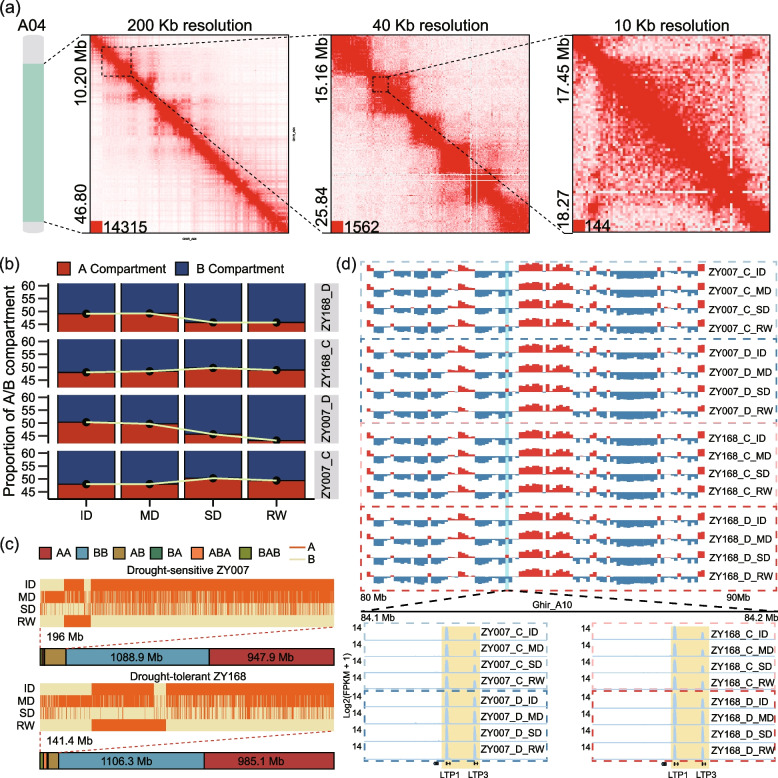
Switching between A/B compartment. **a** Heatmap of chromatin interaction in part of regions of A04 at different resolutions at the ID of ZY007 under control treatment as an example of 3D chromatin map. **b** Proportions of the A/B compartment in two varieties for the four stages under the two treatments. **c** Global dynamic switching of chromatin compartment status. Dark yellow and light yellow represent the A and B compartment, respectively. Heatmaps show bins with status switching between A compartment and B compartment. AB means switching from A compartment to B compartment (including AAAB, ABAB, AABB, ABBB). BA means switching from B compartment to A compartment (including BAAA, BABA, BBAA, BBBA). ABA means switching from A compartment to B compartment and then from B compartment switching to A compartment (including ABBA, ABAA, AABA). BAB means switching from B compartment to A compartment, and then from A compartment to B compartment (including BAAB, BABB, BBAB). **d** The figure above shows a region on chromosome D03 that undergoes drought-induced switching from A compartment to B compartment. The figure below shows the changes in the expression of two genes in the switching region

In addition to transcriptional regulation, emerging researches underscore the impact of environmental stressors on chromatin architecture [18–20]. In order to investigate the changes in chromatin higher-order structure at the 3D genomic level and their potential biological functions in cotton, we performed in situ Hi-C experiments of the 32 samples described above. Ultimately, we obtained 32 Hi-C libraries containing approximately 63.7 billion raw sequences (ranging from 1.37 billion to 5.16 billion). After initial processing, we obtained a total of 12.8 billion valid interactions (ranging from 330 to 480 million; Additional file 2: Table S5). The correlation between the two biological replicates was good (correlation coefficient ranging from 0.93 to 0.97; Additional file 1: Fig. S3a). Therefore, we merged the two biological replicates for further analysis. The resolution of the merged 16 samples can reach 10 kb, with seven samples reaching 5 kb (Additional file 1: Fig. S3b). Chromatin exhibits distinct and obvious structures in the heatmaps at different resolutions, ensuring the feasibility of our subsequent analysis of A/B compartments and TAD (Fig. 4a). Overall, the high-resolution 3D genomic map of cotton in response to drought stress is reliable.

The A/B compartment is an important structure about the chromatin, where the A and B compartment represents active and repressive chromatin state, respectively [26]. We identified the A/B compartments of 16 samples using a 100-Kb resolution interaction matrix. In the control, the A compartment increased and the B compartment decreased as development progressed, while an opposite trend was observed under drought (Fig. 4b). It is noteworthy that in ZY168, the downward trend of the A compartment after rehydration finished, whereas in ZY007, the A compartment continued to decrease after rehydration. Specifically, ZY007 and ZY168 had intervals of 196 Mb and 141 Mb where the A/B compartment switching occurred during drought. From the heatmap, it can also be seen that ZY007 had more regions where the irreversible (not recovered after rehydration) switching from the A compartment to the B compartment occurred (Figs. 4c, S4a). For genes, both varieties showed more genes undergoing switching from the A compartment to the B compartment during drought stress (Additional file 1: Fig. S4b), especially during ID stage (more than 30 genes per Mb underwent switching from the A compartment to the B compartment). Combined with transcriptome data, the gene expression level in the A compartment was significantly higher than that in the B compartment (Additional file 1: Fig. S4c). These genes undergoing compartment switching were more likely to have differential expression than other genes (Chi-squared test, X-squared = 72, *P* < 2.2 × 10^–16^). Among the Module 2 genes identified above, we found two genes, *LTP1* and *LTP3*, within the regions undergoing A/B compartment switching (Fig. 4d). The corresponding gene expression levels showed a significant increase following switching from the B compartment to the A compartment under drought stress. In *Arabidopsis*, many members of the LTP family are induced by drought [27]. These results indicate that under drought induction, some genes are involved in the switching between the A/B compartments, and these switching are related to differential gene expression.

### Dynamic TAD landscape of cotton development

**Fig. 5:**
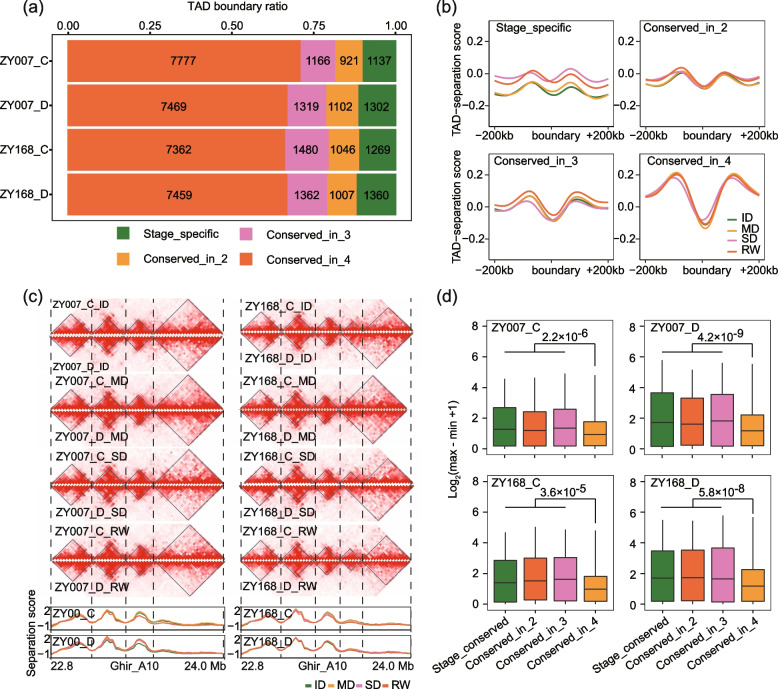
Comparison of TAD boundaries in different stages. **a** The proportion of the four-type TAD boundaries. The green represents stage-specific TAD boundaries, and the yellow, pink, and orange represent TAD boundaries conserved in two, three, and four stages, respectively. **b** TAD separation score of the regions where the boundaries of the four types of TADs are located (200 Kb left and right). **c** An example of a dynamic TAD boundary between stages. The heatmap shows the interaction strength of chromosome 22.8 Mb to 24.0 Mb of A10. The darker the color, the stronger the interaction. The figure below shows the TAD-separation score in this region. **d** The expression dynamics of the genes contained in the four types of TAD boundary regions in the four stages, expression dynamics was expressed using the logarithm of the range of gene expression (FPKM)

TAD is a higher-order chromatin structure that occurs at mega-base resolution, playing a crucial role in maintaining gene expression stability under stress conditions [28]. To explore how TADs change under drought treatment, we identified TAD for 16 samples at 20-Kb resolution. A total of 140,376 TAD structures were detected, with the highest number occurring during SD of ZY168 control treatment (8979) and the lowest during the SD of ZY007 drought treatment (8578; Additional file 1: Fig. S5a). The number of TADs remained relatively constant across different treatments and developmental stages. The expression levels of genes located at TAD boundaries were significantly higher than those within TADs in all samples (Additional file 1: Fig. S5b). Next, we performed iterative comparisons among different stages to construct a “pan-TAD boundary” landscape for four stages in two varieties under two treatments (see “Methods”). After removing redundancy, the pan-TAD boundary sets for the four stages ranged from 11,001 to 11,192. We classified TAD boundaries into four categories: stage-specific, conserved in two stages, conserved in three stages, and conserved in all stages. The percentages of TAD boundaries conserved in four stages were the highest, while the percentages of TAD boundaries conserved in two stages was the lowest (Fig. 5a).

To investigate the characteristics of above four types of TAD boundaries, we examined the insulating index (inferred as TAD-separation score) of each 200-Kb region to the left and right of the TAD boundary. The results showed that more conserved TAD boundaries had lower TAD-separation score (indicating greater stability), while stage-specific TAD boundaries were less stable (Fig. 5b,c). Finally, we combined transcriptome data to examine the differences in gene expression within these four categories of TAD boundary regions. The results showed that genes located within TAD boundaries conserved across all four stages had the smallest expression differences among the four stages, whereas genes within dynamic TAD boundaries showed larger expression differences over time (Fig. 5d). This indicates that the higher-order chromatin structure of plants has a certain degree of plasticity across different developmental stages and disruption of TAD boundaries are associated with dynamic changes in gene expression.

### Disruption of TAD structure induced by drought

**Fig. 6:**
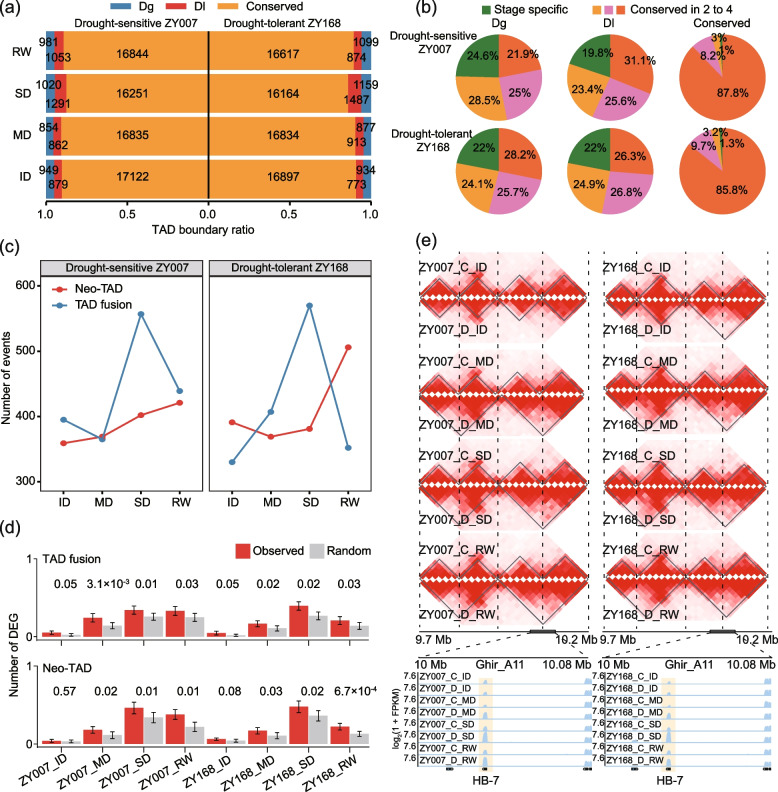
TAD changes under drought induction. **a** Proportions of the three types of TAD boundaries relative to the control in the four stages of the two varieties (dg drought gain, dl drought loss). **b** The ratio of the three types of TAD boundaries in dynamic level. Green represents stage-specific TAD boundaries; yellow, pink, and orange represent conserved in two, three, and four stages, respectively. **c** Number of two TAD disruption events (TAD fusion and neo-TAD). **d** The number of DEGs in the TAD boundary region related to TAD disruption events. Here, 10 Kb on each of the left and right sides of the TAD boundary are designated as the TAD boundary region. **e** A drought-induced TAD fusion event. The heat map shows the chromatin interaction frequency in this region, and the darker the color, the higher the interaction frequency. The yellow highlight shows a drought-induced up-regulated gene within the TAD boundary

To investigate how TAD boundaries change under drought stress, we compared TAD boundaries between control and drought-treated conditions. We classified TAD boundaries into three categories: TAD boundaries conserved under drought (conserved), TAD boundaries formed under drought (dg: drought gain), and TAD boundaries lost under drought (dl: drought loss; see “Methods”). Among these three types of boundaries, conserved TAD boundaries were the most prevalent (85.9% to 90.74%), and their proportion decreased with increasing severity of drought stress, reaching the lowest point in SD (Fig. 6a). After rehydration, the proportion of conserved TAD boundaries increased from 85.9 to 89.4% in ZY168, higher than ZY007 (from 87.5 to 89.2%). These results indicate that ZY168 had a stronger drought resistance reflected at the level of chromatin structure.

We investigated the characteristics of the above three types of TAD boundaries. Firstly, we observed that TADs with conserved TAD boundaries were significantly larger than the TADs with dg or dl boundaries (Additional file 1: Fig. S5c). In addition, more than 85% of conserved TAD boundaries under drought were also conserved between stages (87.8% for ZY007 and 85.8% for ZY168), whereas only 21.9% to 31.1% of dg and dl boundaries were conserved between stages (Fig. 6b). This indicates that dg and dl boundaries only occur during specific stages rather than being conserved between stages. We observed the distribution of these three types of TAD boundaries throughout the whole genome and found, interestingly, that dg was preferentially located in telomere regions during the ID and MD stages, while dl was preferentially distributed in chromatin arms. This distribution preference changed during SD and RW (Additional file 1: Fig. S5d).

We focused on two important TAD change events: TAD fusion and Neo-TAD (see “Methods”) [29]. We identified a total of 6597 TAD change events, including 3407 TAD fusions events and 3190 Neo-TAD events (Additional file 2: Table S6). Notably, both varieties exhibited significantly more TAD fusion events in SD than in the MD (Fig. 6c). After rewatering, ZY168 showed significantly more Neo-TAD events than ZY007 (506 vs. 421, Fig. 6c). In conjunction with gene expression, we found that both types of TAD changes had a significantly higher number of DEGs than expected, indicating that these two important TAD changes had some impact on gene expression (Fig. 6d). Except for RW, the A_t_ subgenome had fewer TAD change events than the D_t_ subgenome in the other three time points, which could be associated with the stronger expression suppression of the D_t_ (Additional file 1: Fig. S5e). Combining with module 2 previously screened as significantly related to drought resistance, we identified a gene *HB-7* that is located at a dg boundary following a TAD fusion event (Fig. 6e). This gene is also up-regulated in response to drought and is a core gene in module 2 (Fig. 3b). In *Arabidopsis*, this gene encodes a transcription factor of the HOMEOBOX family, which is dependent on abscisic acid for transcriptional regulation of downstream genes and plays a role in the signal transduction pathway of drought response [25]. All of these findings suggest that TAD structures undergo certain changes under drought induction, and these changes may be associated with the transcriptional regulation of drought-related genes.

We investigated the dg and al hotspots between two varieties under drought (see “Methods”). We identified a total of 165 and 212 dg and dl hotspots in the two varieties, respectively, with an average of 8.8 and 9.2 changed TAD boundaries per hotspot (Additional file 1: Fig. S5f; Additional file 2: Table S7). With the exception of the ID stage, ZY168 had more dg hotspots than ZY007 in all other stages. Notably, dl hotspot numbers showed a significant increase during the SD and decreased significantly after rehydration in both varieties. However, the reduction of dl hotspots was greater in ZY168 (from 73 to 13) than in ZY007 (from 54 to 31; Additional file 1: Fig. S5f). Analysis of the two varieties revealed that 19.4% of dg hotspots (32/165) and 33.0% of dl hotspots (70/212) were shared. These findings suggest that 3D structures of the two varieties were greatly disrupted in SD following a large number of TAD boundary loss, and after rehydration, the TAD structure of drought-tolerant ZY168 was more resilient than drought-sensitive ZY007.

### Screening of drought-induced genes associated with 3D genomic variation

To facilitate the study of functional genes related to cotton drought resistance, we summarized and screened for a set of genes (referred to as target genes) that are both related to 3D chromatin structure and induced by drought stress. We employed the following filters to identify genes of interested supported by three pieces of evidence: (1) differentially expressed genes induced by drought stress in the two varieties (ZY007 and ZY168, with 22,606 and 19,552 genes, respectively, Fig. 2a); (2) genes in module 2 (506 in total, Fig. 3a); and (3) genes associated with TAD boundary changes occurring under drought induction (specifically referring to the TAD boundaries of dg and dl, with 13,641 genes in ZY007 and 15,436 in ZY168). We then took the intersection of these three gene sets in each variety separately to obtain the target genes for each variety. Finally, we compared the target genes between the two varieties and took the intersection of the target genes from both varieties.

**Fig. 7:**
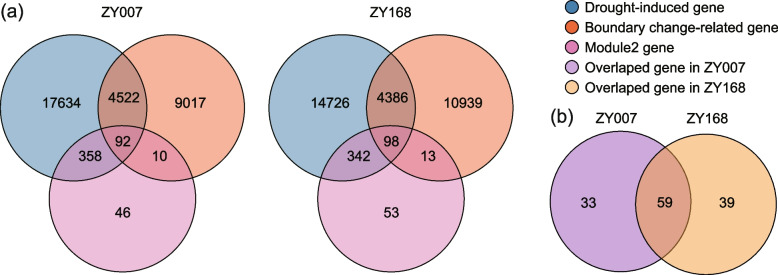
A filter for genes associated with 3D chromatin structure and induced by drought. **a** The left and right figures represent the results of genes of ZY007 and ZY168, respectively. The blue, red, and pink circles represent drought-induced genes, drought-altered TAD boundary-related genes, and module 2 genes, respectively. **b** The intersection of the target genes in the two varieties. The purple and yellow circles represent the target genes of ZY007 and ZY168, respectively

Using our filters, we identified 92 and 98 target genes that were supported by multiple pieces of genomic evidence in ZY007 and ZY168, respectively (Fig. 7a). Among these target genes, 59 were present in both varieties, while ZY007 and ZY168 had 33 and 39 specific target genes, respectively (Fig. 7b). These target genes include several stress-responsive genes such as *PP2C* [30], *GST8* [31], *PR*, and *HSP20* [32]; protein kinases and phosphatase genes involved in stress response such as *CIPK6* [33], *PFK4* [34], and *HAI2* [10]; transport-related genes such as *PDR*, *NRT2.4* [35], and *ANN5* [36]; and 17 TF encoding genes such as *TGA4* [37], *WRKY23* [38], and *HB-7* [25] (Additional file 2: Table S8). Overall, we identified a series of genes both related to 3D chromatin structure and induced by drought stress, which are closely related to cotton response to drought stress.

## Discussion

### The cotton accession ZY168 displays resilience to drought

Previous studies on plant stress have primarily focused on investigating individual genes or at the genetic and transcriptomic levels. Our research approach takes a phenotype-first perspective and utilizes transcriptomics and 3D genomic analysis to better understand the plant’s response to drought. In the SD stage, we observed noticeable differences in phenotypes between ZY168 and ZY007. The leaves of ZY007 wilted more severely, while ZY168 gradually recovered after rewatering. Transcriptome analysis revealed that the gene expression profiles of ZY168 and ZY007 under RW were significantly different. Specifically, the samples of ZY168 clustered with the control group, whereas the samples of ZY007 clustered with the samples under sustained drought stress. Furthermore, the expression level of drought-induced genes returned quickly to the control state after rehydration in ZY168, indicating strong recovery and drought resistance.

Our analysis of the chromatin hierarchical structure also revealed notable differences between ZY168 and ZY007. After rehydration, 82% of the drought-induced switching compartments were restored in ZY168, compared to only 57% in ZY007. Moreover, the reduction of TAD fusion events in ZY168 was significantly higher than that in ZY007 after rehydration, with ZY168 experiencing a reduction from 570 to 352, compared to ZY007’s reduction from 557 to 439. Previous studies have shown that the establishment of TAD boundaries is conducive to more orderly expression in cells, and the loss of TAD boundaries may lead to dysregulation of gene expression [39, 40]. After rehydration, ZY168 had more drought-gained boundaries and less drought-lost boundaries than ZY007, which may reflect that gene expression regulation in ZY168 may be more orderly to a certain extent. The ability to recover after rehydration is one of the important manifestations of drought tolerance. We conclude that cotton germplasm with strong drought tolerance exhibits greater gene expression and chromatin hierarchical structural plasticity, which could contribute to its ability to recover after rehydration.

### Asymmetric activity of subgenomes in response to drought

Allopolyploids, like allotetraploid cotton, often exhibit coordinated regulation of multiple subgenomes to control important agronomic traits [41]. In this study, we investigated the similarities and differences in responses to drought stress between the two subgenomes in three aspects. First, under drought induction, the two subgenomes tended to exhibit consistent differential expression (Fig. 2d). Interestingly, as drought intensified, the expression of homoeologous genes gradually shifted from one exhibiting differential expression to both subgenomes exhibiting differential expression in the same direction. Second, drought treatment exacerbated the stage dynamics of expression differences among homoeologous genes (Fig. 2e), indicating that although the two subgenomes tended to show differential expression in the same direction, the magnitude of the differential expression was not necessarily the same. Third, under drought treatment, the expression of both subgenomes was suppressed, but the suppression was stronger in the D_t_ subgenome. As drought severity increased, the level of differential expression suppression in both subgenomes gradually decreased in ZY168, but gradually increased in ZY007, which could be associated with more TAD change events in the D_t_ subgenome (Fig. 2g). Previous studies have shown that cotton resistance-related genes are mostly concentrated in the D_t_ subgenome, so the weaker suppression of the D_t_ subgenome of ZY168 may be the reason for its stronger resistance [5]. Most of the previous studies on the asymmetric expression of subgenomes just focused on the association analysis of quantitative traits in the allopolyploid plant [42]. This study provides valuable insights into how two subgenomes respond to environmental stress in plants.

#### Chromatin hierarchical structure changes in response to drought in cotton

In this study, we utilized in situ Hi-C technology to construct chromatin interaction maps for two varieties under two treatments at four stages. Compared to previous studies on the 3D genome of plants under stress, our dataset represents an unprecedented scale, and the inclusion of multiple time points ensures that we can investigate the dynamic changes of chromatin structures in response to drought stress [19, 20].

In terms of A/B compartment switching, we found that as drought time lengthened and severity increased, there was an increase in the conversion from A to B compartments, indicating that the expression of the whole genome was suppressed under drought stress. This is consistent with the pattern of A/B compartment switching under heat stress [19, 20]. We focused on two important TAD changes: TAD fusion and the Neo-TAD. We found that both dynamic TAD structural changes between stages and TAD changes induced by drought could lead to greater differential gene expression (Fig. 6d, e). Among the genes related to these two TAD changes, we identified a TF encoding gene *HB-7* that is upregulated by drought induction. This gene is located in the TAD fusion event and is also a core gene in module 2. Previous studies on *Arabidopsis* have also demonstrated the involvement of this gene in drought response (Fig. 6e) [25].

Based on transcriptome and 3D genome analyses, we identified a total of 131 genes that are induced by drought and associated with 3D genomic variation including stress-related TFs. In-depth analysis of these genes will help us better understand the biological response mechanisms of cotton under drought conditions.

## Conclusions

Rapid advances have been made in understanding the regulatory mechanisms of three-dimensional (3D) genomics on abiotic stress response in plants. In the current study, combined with transcriptional analysis, a high-resolution drought-related 3D genomic map of two varieties that exhibit differences on drought tolerance and recovery capacity (drought-sensitive and drought-tolerant) was constructed. We elucidated the mechanisms of cotton drought response and the differences between the two varieties at both transcriptional and 3D genomic levels. The D_t_ subgenome was subject to stronger transcriptional inhibition than the A_t_ subgenome under drought stresses, and we highlighted that the dynamics of A_t_ subgenome bias expression under drought stresses in these two varieties might lead to their differences on drought tolerance and recovery capacity. The dramatic patterns in higher-order chromatin structures including A/B compartment switching and TAD changing events under drought stress were well-illustrated, and more than 6000 TAD variation events were identified between the two varieties under drought stresses. Finally, 131 genes related to chromatin organization variations and induced by drought were identified.

## Methods

### Cotton varieties and drought treatment

Two cotton varieties, Junmian 1 (ZY007) and Wankangmian 9 (ZY168), were chosen based on their significant differences in drought tolerance [21]. Seeds of the two varieties were germinated in a moist and dark environment at a temperature of 30℃. The seedlings with uniform growth were then transplanted into pots with a consistent soil weight. The experimental conditions included a temperature of 30℃, a relative humidity of 50%, and a light–dark cycle of 8 h of darkness and 16 h of light (PPF = 120 μmol/s). The drought treatment started at the developmental stage when plants had 4 leaves and 1 apical meristem.

The drought treatment began after saturating the plants with water for 24 h. The control group was watered with 1 L of water per 40 pots once a day, while the treatment group was not watered; 24 h after watering of the control group, 10 randomly selected pots from each variety in the treatment group and control group were weighed to determine their relative water content of the plant. Phenotypic data were collected using a self-developed automatic device to capture eight side views of each plant (captured every 45°) for analysis of plant drought severity [21].

On the fourth day of drought treatment (4 day), cotyledon edges of treated plants mildly softened (initial drought stage, ID). On the sixth day (6 day), cotyledons wilted completely (mild drought stage, MD). On the ninth day (9 day), the first leaf wilted (severe drought stage, SD). On the tenth day, all leaves substantially wilted. On the 11th day, all plants were re-watered to saturation (re-water stage, RW). During the ID, MD, SD, and RW stage, ten randomly selected plants from each variety in the treatment group and control group were collected, with the second leaf from the top being sampled. The experiment included two biological replicates.

### Transcriptome and in situ Hi-C experiments

Tissue samples were rapidly ground in liquid nitrogen. Total RNA was extracted using the HiPure Universal RNA Mini Kit (Magen, R4165-02), and an RNA-seq library was constructed using the VAHTS Universal V8 RNA-seq Library Prep Kit for MGI (Vazyme, NRM605-01). Sequencing was performed using a MGISEQ-T7 platform.

The in situ Hi-C methodology described previously was improved [43]. Tissue samples were rapidly ground in liquid nitrogen. NI buffer containing 1% (v/v) formaldehyde was added at room temperature and quenched with glycine buffer. The sample was filtered through double layers of miracloth and a single layer of nylon membrane to obtain the cell nuclei. Samples were digested with *DpnII* restriction endonuclease, labeled with biotin-14-dCTP, and ligated. Approximately 1 μg of DNA sample was sonicated to fragment the DNA, end repaired, and 300 to 500 bp fragments screened. Biotin-labeled DNA fragments were purified using Dynabeads MyOne Streptavidin T1 magnetic beads (Invitrogen, 65,602). Library construction was performed using the VAHTS Universal DNA Library Prep Kit for MGI (Vazyme, NDM607-01). Sequencing was performed using the MGISEQ-T7 platform.

### Genome adjusting depending on resequencing

BWA was used to map the genomic resequencing data of the two varieties generated in the previous study to the high-quality TM-1 genome with default parameters [44]. After sorting with SAMtools [45], picard (version 2.23.9) was used to remove PCR duplication (https://github.com/broadinstitute/picard). GATK (version 4.1.9.0) and was used to convert bam file to gvcf file (parameter: -ERC GVCF -stand-call-conf 30) and perform SNP calling on gcvf with default parameters (https://github.com/broadinstitute/gatk). Finally, keeping the genome size and annotation unchanged, the genotypes of TM-1 were replaced with the genotypes of the two varieties to obtain the adjusted genomes of the two varieties.

### Calculation of gene expression

Firstly, keeping the genome size and annotation unchanged, the genotypes of TM-1 were replaced with the genotypes of the two varieties to obtain the adjusted genomes of the two varieties. After obtaining the sequencing data, we used Trimmomatic to filter the data [46]. HISAT2 was used to compare the filtered high-quality data to the adjusted genomes of the two varieties. SAMtools was used for format conversion and sorting, and StringTie was used to calculate the gene expression level (FPKM) of 32 samples under the default parameters [47, 48]. We used the averaged FPKM of two replicates as the gene expression. During the whole developmental stages, genes with differential expression in at least one stages relative to the control and in the same direction of differential expression (both up-regulated or both down-regulated) during the whole developmental stages were defined as drought-induced genes.

### Gene differential expression and enrichment analysis

FeatureCounts was used to calculate the raw count of each gene in 32 samples with default parameters, and then the raw count matrixes were constructed [49]. DESeq2 was used to calculate the differentially expressed genes (DEGs) under the drought treatment relative to the control [50]. Enrich-analysis.pl script was used to perform GO enrichment analysis on the DEGs corresponding to each stage of each variety (Fisher’s exact test; https://github.com/xukaili/Enrich-analysis.pl).

### Subgenomic expression analysis

We performed an all-vs-all blastp comparison of two subgenomes of the TM-1 genome using blastp (E-value < 1 × 10^–10^, -v 5, -b 5). The results of blastp were used to identify homoeologous genes between subgenomes by the MCScanX package [51]. Subgenomic expression analysis mainly had the following three considerations.

Firstly, relative to the control, we focused on the change direction of the two genes of the homoeologous gene pairs (up- or down-regulation). Homoeologous genes were classified into three categories based on their expression patterns. The first category includes genes that did not show differential expression or showed the same patterns of differential expression (common). The second category includes genes where only one homolog showed differential expression (single). The third category includes genes where both homologs showed differential expression, but in opposite directions (opposite).

Secondly, we focused on dynamic changes in expression differences between two homoeologous genes in four stages under different treatments of two varieties. The homoeologous gene pairs were classified into three categories. The first and second categories included genes where the expression of the A_t_/D_t_ subgenome was consistently higher than that of the D_t_/A_t_ subgenome across all four stages (stableAtbias and stableDtbias). The third category included genes where the direction of expression bias between the two homologs changed across the four stages (dynamic).

Thirdly, we specifically examined homoeologous gene pairs that showed changes in expression bias under drought conditions compared to control conditions in the two varieties. We used a bias change index to measure the strength and direction of bias at four stages. The index assigned values: Atbias as 1, Dtbias as − 1, and nobias as 0. Subtracting the bias index under control from drought yielded the difference, indicating the bias change index for each gene pair. The sum of these indices represented the whole-genome bias change under drought, where a positive value indicated bias towards the A_t_ subgenome and a negative value indicated bias towards the D_t_ subgenome. Moreover, the absolute value of the index represented the magnitude of the bias, with larger values indicating stronger bias.

### Construction of gene co-expression network

We used the gene expression FPKM matrix of 32 samples (only containing DEGs) to construct the gene co-expression network, utilizing the WGCNA package [52]. The steps involved: filtering genes with expression > 0.5 in at least one sample, retaining genes with median absolute deviation > 0.01 in the top 75%, removing missing values, identifying outliers, conducting soft threshold (power) screening, and constructing co-expression network. We only analyzed the co-expression relationship with weight greater than 0.2. Cytoscape was used to visualize the co-expression network [53].

### Preliminary processing of Hi-C data

Raw data were filtered using Trimmomatic (version 0.32) to obtain high-quality Hi-C data. Then, using the HiC-Pro software (version 2.7.1) under the default parameters, each Hi-C data was mapped to the two varieties-adjusted genomes to obtain the Hi-C interaction matrix [54]. In addition, 20-Kb and 100-Kb interaction matrices were converted to.cool and.h5 format files using HiCExplorer [55], and Juicer pre command was used to convert the interaction matrix to a.hic file [56]. We repeated the above steps after merging the two replicates, and the merged interaction matrix was used for further analysis.

### Identification of A/B compartments and TADs

We primarily identified A/B compartments using the cword software with a 100-Kb raw interaction matrix (https://github.com/dekkerlab/cworld-dekker). The steps involved sparseToDense.py to create a paired matrix with added row names, followed by matrix2loess.pl to generate a zScore standardized symmetric matrix. Finally, the matrix2EigenVectors.py script of cword is used to identify the A/B compartment. In the resulting file, bins with eigenvalues greater/less than 0 are A/B compartments. We used the 20-Kb interaction matrix to identify the TAD by TADLib [57]. First, HiCExplorer was used to convert the 20-Kb original interaction matrix into a.cool format file, and then the domaincaller function in TADLib was used to identify the TAD. This function is mainly based on the TAD identification method based on directional index (DI) [58]. In addition, we also used the hicFindTADs program in HiCExplorer to calculate the TAD-separation score (an index of the insulation strength) at 20 Kb. The regions at both ends of the TAD are referred to TAD boundary regions.

### TAD comparison

We defined the TAD boundary region as a region of 40 Kb on both sides of the TAD boundary (80 Kb in total). Then, the intersectBed function in BEDTools was used to intersect the two boundary regions [59]. Two TAD boundaries were defined as conserved TAD boundaries if their regions had an intersection of at least 1 bp. If the four TAD boundaries of two TADs are conserved in pairs, then these two TADs are defined as conserved TADs. The comparisons of TAD were mainly divided into three aspects.

Firstly, we performed the comparison between stages and constructed a “pan-TAD boundary” map using an iterative approach, similar to pan-genome construction. For instance, in constructing the “pan-TAD boundary” under ZY007’s control treatment, we first compared each stage with the others. Next, we included all TAD boundaries from the ID. Afterward, we incorporated TAD boundaries specific to MD relative to ID, then those specific to SD relative to both ID and MD. Finally, we added TAD boundaries specific to RW in ID, MD, and SD. We then reintroduced each TAD boundary from this set back into the TAD boundary comparison results to obtain the conservative conditions of the “pan-TAD boundaries” for all four stages.

Secondly, we performed the comparison of drought and control treatment. Here we mainly focused on two common TAD change events, TAD fusion, and Neo-TAD [29]. TAD fusion is defined as the merger of two or more TADs in the control into one TAD during drought. Neo-TAD is defined as the splitting of one TAD in the control into two or more TADs during drought. We focused on genes within 20 Kb (40 Kb total) on either side of the TAD boundary. We defined the region with more than 8 drought gain (dg) or 8 drought loss (dl) TAD boundaries within 10 Mb as hotspot regions of dg and dl and merged consecutive hotspot regions into one hotspot region.

Thirdly, the comparison between the two varieties. We used the following strategy to construct the “pan-TAD boundary” of 16 samples (two varieties, two treatments, and four stages). Firstly, we found the best one-to-one correspondence between the four boundary sets (two varieties and two treatments). Then, we uniformed each member of the above 4 sets and used the join function in the JCVI package to construct the “pan-TAD boundary” of 16 samples with one set (all 4 sets should be used as a reference) [60]. Finally, the results from different references were merged to construct the final “pan-TAD boundary” set.

### Data visualization

Here, we mainly used.hic files and.cool files to visualize the heatmap by the offline version of Juicebox (version 1.11.08; https://github.com/aidenlab/Juicebox) and TADLib. For the visualization of gene expression and gene structure, bamCoverage of deepTools (version 3.5.0) was used to convert the.bam file into a.bw file [61], the configuration file required by pyGenomeTracks was generated, and finally, pyGenomeTracks was used for visualization [62]. TAD variation hotspots were visualized using Circos [63].

### Supplementary Information

Additional File 1: Figs S1-S5.Fig. S1 Transcriptome correlation and cluster analysis. Fig. S2 Differentially expressed gene identification and subgenomic expression bias analysis. Fig. S3 Hi-C data resolution and correlation between biological replicates. Fig. S4 Compartment switching correlation analysis. Fig. S5 Analysis of TAD variation induced by drought.

Additional File 2: Tables S1-S8. Table S1. Summary of RNA-seq reads mapping. Table S2. Differential expression directions of all DEGs (drought vs control). Table S3. GO enrichment of genes in module 2. Table S4. Genes and annotations in module 2. Table S5. Summary of Hi-C reads mapping. This information was obtained using the HiC-Pro software. Table S6. Summary of all TAD change events including TAD fusion and Neo-TAD. Table S7. Hotspots of TAD changes including dg and dl. Table S8. Annotation of drought-induced gene associated with chromatin 3D structural changes in ZY007 and ZY168.

## Data Availability

All raw sequencing data generated in this paper have been deposited into the National Center for Biotechnology Information database (BioProject ID: PRJNA987406) [64].

## Abbreviations

Hi-C: High-throughput chromosome conformation capture
ID: Initial drought
MD: Mild drought
SD: Severe drought
RW: Re-water
TAD: Topologically associating domain
WGCNA: Weighted correlation network analysis
DEG: Differentially expressed gene
GO: Gene Ontology
