# Supplementary material for: Drought response revealed by chromatin organization variation and transcriptional regulation in cotton
Source: BMC Biol. 2024 May 20;22:114. doi: 10.1186/s12915-024-01906-0 (PMC11103878; doi:10.1186/s12915-024-01906-0)
Supplement: Supplementary file 1 — Additional File 1: Figs S1-S5.Fig. S1 Transcriptome correlation and cluster analysis. Fig. S2 Differentially expressed gene identification and subgenomic expression bias analysis. Fig. S3 Hi-C data resolution and correlation between biological replicates. Fig. S4 Compartment switching correlation analysis. Fig. S5 Analysis of TAD variation induced by drought. [file 12915_2024_1906_MOESM1_ESM.pdf]

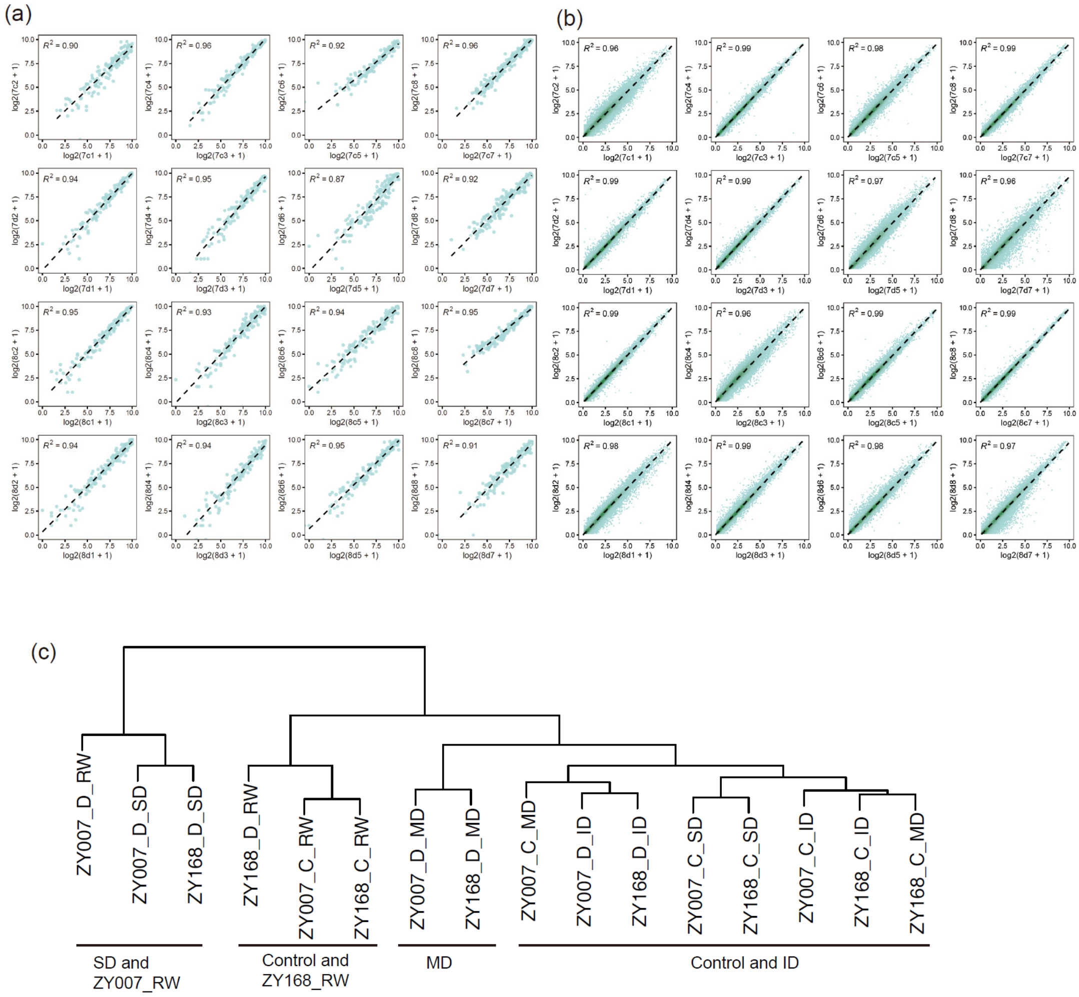

Fig. S1 Transcriptome correlation and cluster analysis.

(a-b) Correlation between biological replicates of transcriptome data based on the read counts (a) and gene expression (b). (c) Clustering results of RNA-seq from 16 samples. 7 and 8 represent ZY007 and ZY168, C and D represent normal water supply and drought stress condition, respectively. ID, MD, SD and RW represent the initial drought, mild drought, severe drought and rewater, respectively.

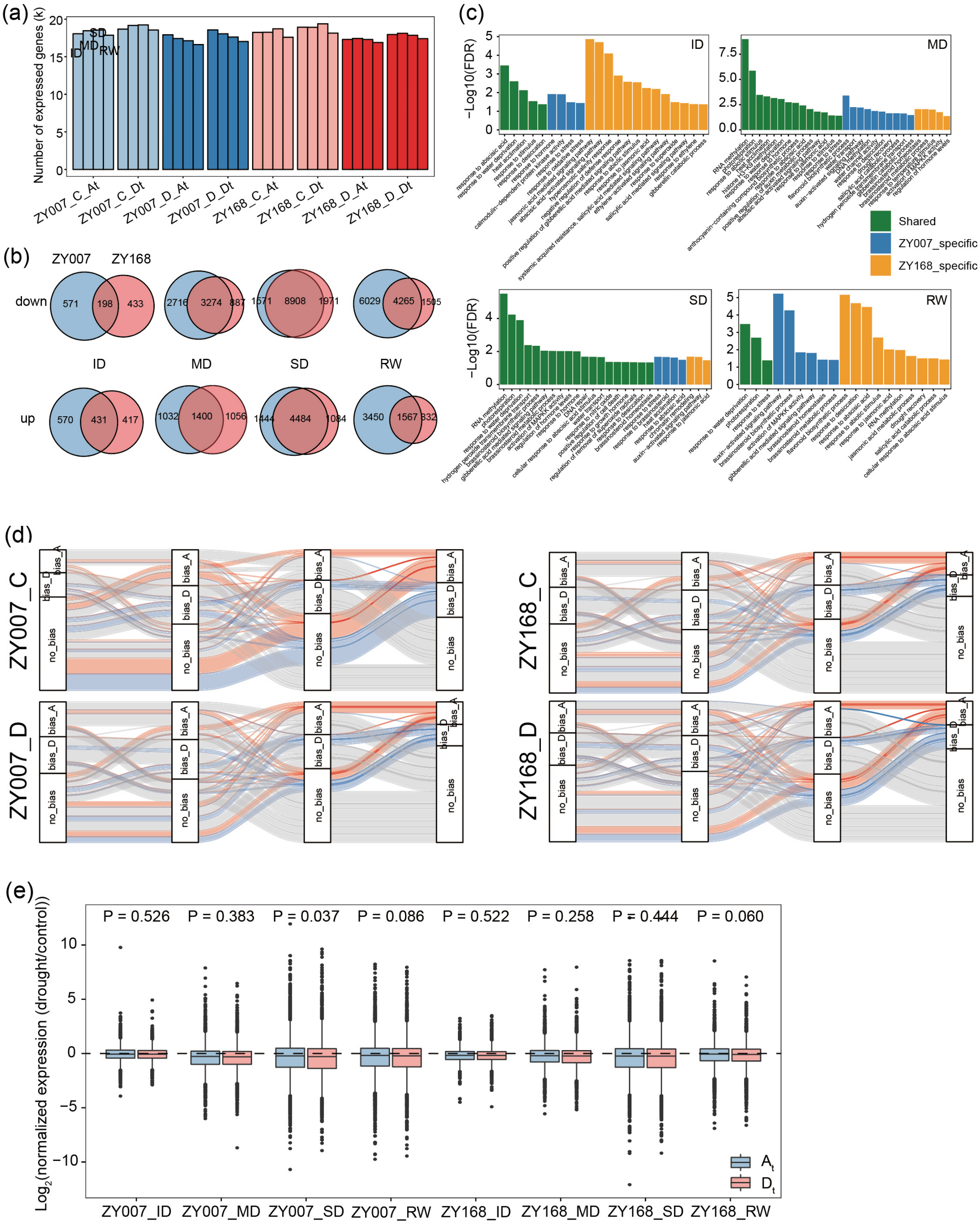

Fig. S2 Differentially expressed gene identification and subgenomic expression bias analysis.

(a) The number of expressed genes in sub-genome A_t and D_t under different stress treatments and stages. (b) The number of DEGs in the four stages of the two samples, with down-regulated expression in the top row and up-regulated expression in the bottom row, and ZY007 and ZY168 in blue and red, respectively. (c) GO enrichment results of DEGs. Green, blue and yellow represent pathways shared by the two cultivars, ZY007-specific pathways and ZY168-specific pathways, respectively. (d) Detailed

presentation of the expression bias of dynamic bias expressed homoeologous genes under four conditions. (e) Corresponding expression levels of genes with changed expression bias induced by drought (Two-sided Wilcoxon rank sum test).

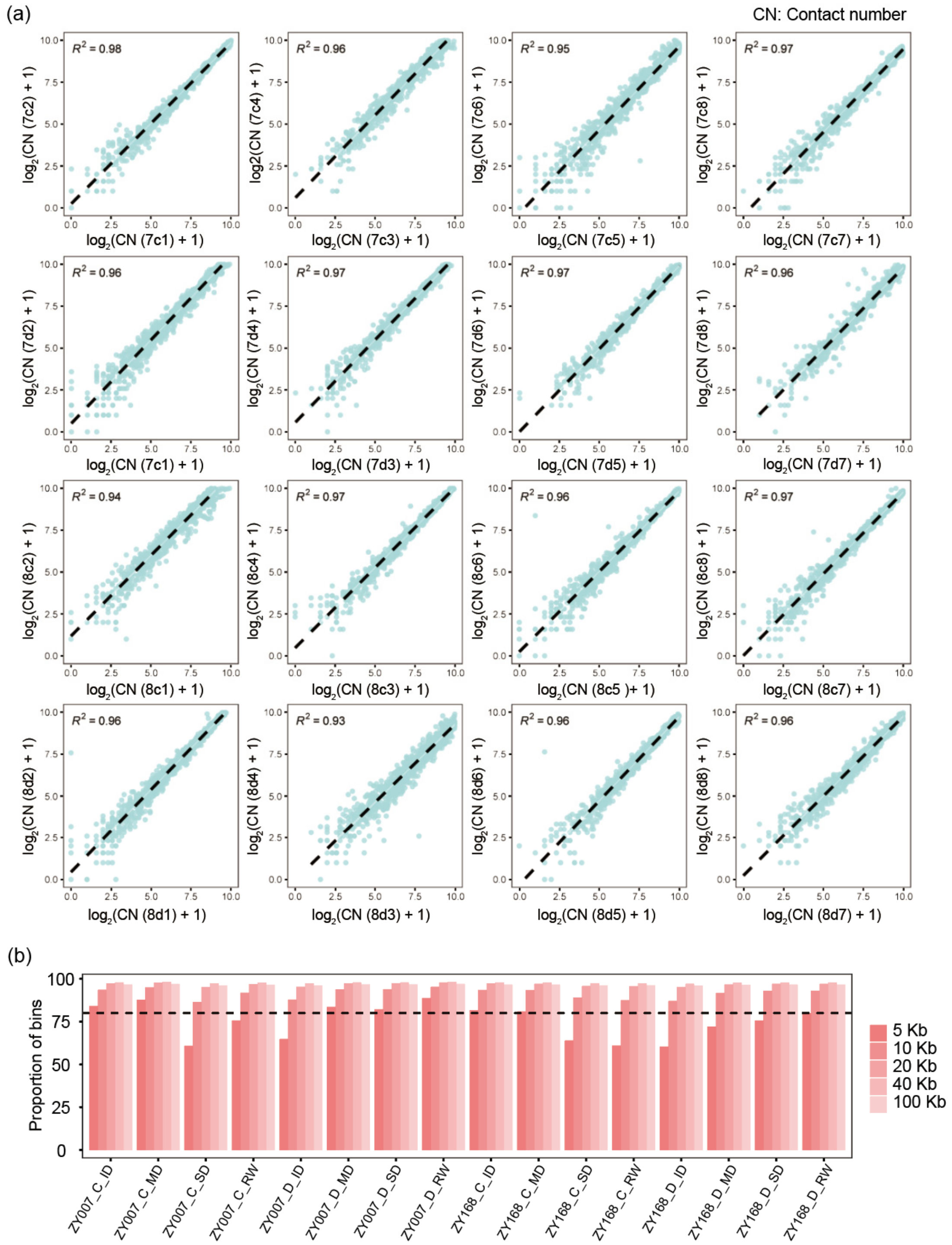

Fig. S3 Hi-C data resolution and correlation between biological replicates.

(a) Correlation of Hi-C data between biological replicates. (b) The proportion of bins with more than 1000 interactions in the total bins, the auxiliary line is 80%, and greater than 80% is the resolution that can be achieved at different resolutions.

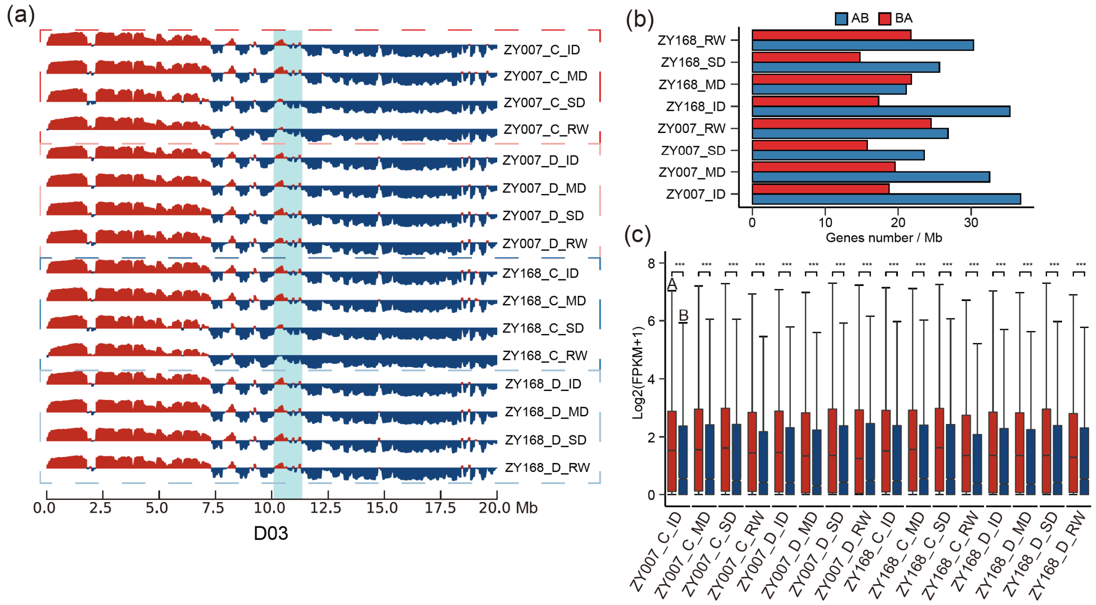

Fig. S4 Compartment switching correlation analysis.

(a) The figure shows a region on chromosome D03 that undergoes drought-induced switching from A compartment to B compartment. (b) Gene densities related to the two types of compartments switching induced by drought compared to control. (c) Comparison of expression of genes in A and B compartments in 16 samples (Two-sided Wilcoxon rank sum test, ***P< 0.001).

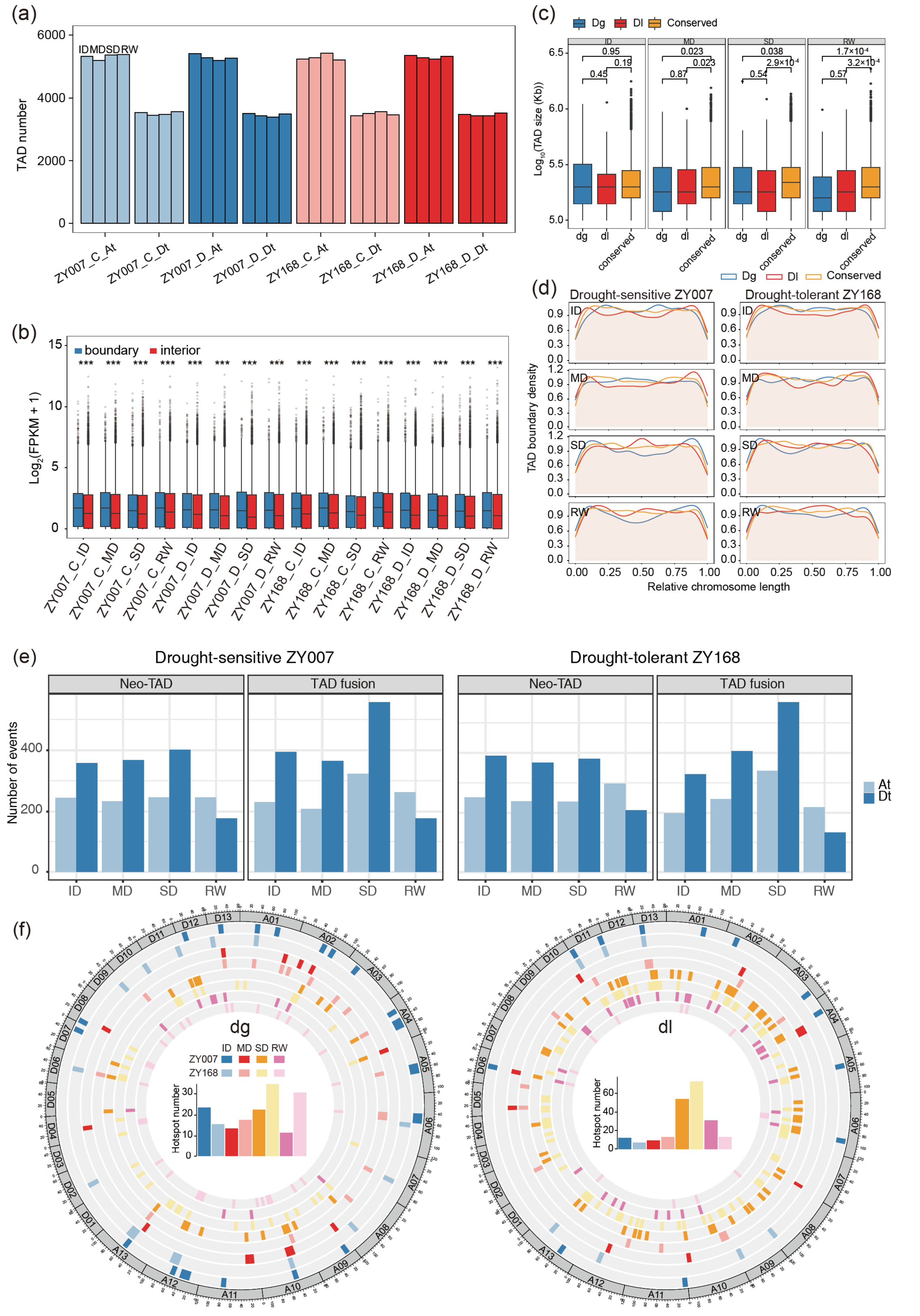

Fig. S5 Analysis of TAD variation induced by drought.

(a) Number of TAD in the A_t and D_t subgenomes in 16 samples. (b) Comparison of expressions between genes distributed in the boundary and interior regions of TAD in

16 samples. (c) Display of the TAD size where the TAD boundaries of the three types. (d) The density curves of the three types of TAD boundaries on the genome. Before calculating the density, we normalized the position of the TAD boundaries according to the chromosome size. (e) The number of two TAD change events between the two subgenomes in four stages of two varieties. (f) The left and right figures show the dg and dl hotspots, respectively. The blue, red, orange, and pink colors represent ID, MD, SD, and RW stages, respectively. The darker and lighter of the same colors represent ZY007 and ZY168, respectively. The histogram in the circle chart shows the number of hotspots, and the color is consistent with the circos figure.
